# Systematic identification of 3′-UTR regulatory elements in activity-dependent mRNA stability in hippocampal neurons

**DOI:** 10.1098/rstb.2013.0509

**Published:** 2014-09-26

**Authors:** Jonathan E. Cohen, Philip R. Lee, R. Douglas Fields

**Affiliations:** Section on Nervous System Development and Plasticity, The Eunice Kennedy Shriver National Institute of Child and Human Development, National Institute of Health, Building 35, Room 2A211, Bethesda, MD 20892-3714, USA

**Keywords:** 3′-UTR, activity-dependent plasticity, miRNA, mRNA stability

## Abstract

Ongoing neuronal activity during development and plasticity acts to refine synaptic connections and contributes to the induction of plasticity and ultimately long-term memory storage. Activity-dependent, post-transcriptional control of mRNAs occurs through transport to axonal and dendritic compartments, local translation and mRNA stability. We have identified a mechanism that contributes to activity-dependent regulation of mRNA stability during synaptic plasticity in rat hippocampal neurons. In this study, we demonstrate rapid, post-transcriptional control over process-enriched mRNAs by neuronal activity. Systematic analysis of the 3′-UTRs of destabilized transcripts, identifies enrichment in sequence motifs corresponding to microRNA (miRNA)-binding sites. The miRNAs that were identified, miR-326-3p/miR-330-5p, miR-485-5p, miR-666-3p and miR-761 are predicted to regulate networks of genes important in plasticity and development. We find that these miRNAs are developmentally regulated in the hippocampus, many increasing by postnatal day 14. We further find that miR-485-5p controls NGF-induced neurite outgrowth in PC12 cells, tau expression and axonal development in hippocampal neurons. miRNAs can function at the synapse to rapidly control and affect short- and long-term changes at the synapse. These processes likely occur during refinement of synaptic connections and contribute to the induction of plasticity and learning and memory.

## 1. Introduction

Long-term modifications to synaptic connections through neural activity-dependent plasticity require gene transcription and translation [1]. The contribution of post-transcriptional gene regulation to activity-dependent neural development and synaptic plasticity is not well understood. Rapid, post-transcriptional changes that may occur at the synapse following synaptic activity act through mRNA transport from the nucleus to axonal and dendritic compartments [2,3], local translation of axonal- and dendritically targeted mRNAs [4], and control of transcript stability [5].

We hypothesized that neural activity may specifically regulate post-transcriptional gene expression through rapid regulation of mRNA stability at synaptic sites in part due to the very polarized structure of neurons into axonal, somatic and dendritic compartments (which are further compartmentalized into more than 10 000 post-synaptic terminals per cell). Axonal and dendritic transcripts have been identified that are transported to pre- and post-synaptic terminals where they are regulated by local translation [6–8] and contribute to synaptic plasticity [9,10]. However, few studies have identified transcripts that are regulated through mRNA stability mechanisms [11–13] and this is complicated by the spatial distribution of transcripts in the cytoplasmic compartments. Moreover, mRNA stability at the synapse can be regulated through complex interactions between many hundreds of RNA-binding proteins (RNA-BPs) [14,15], recognition motifs within the 3′-UTR of synaptic transcripts, very few of which have been identified or characterized [16–18]. There are potentially thousands of non-coding regulatory RNAs [19,20]. These factors have made it challenging to identify specific mechanisms that may control mRNA stability in an activity-dependent and synapse-specific manner. Furthermore, many high-throughput approaches involving transcriptome analysis either through microarray, RNAseq, or RNA–protein interactions, may not identify specific mechanisms that function in this manner [21,22].

**Figure 1.**
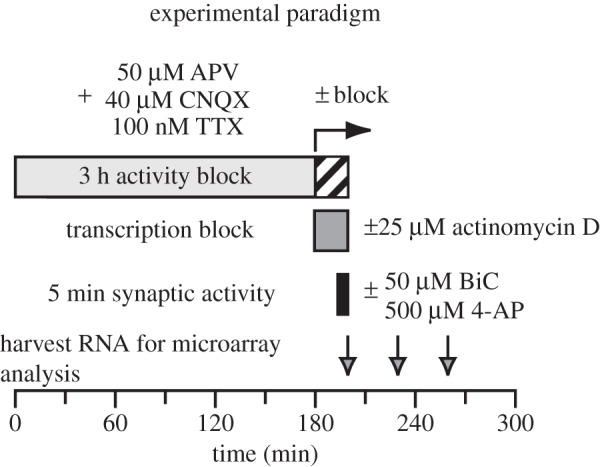
Activity-dependent, post-transcriptional gene regulation experiment. Cultured hippocampal neurons were pre-incubated with an inhibitory drug cocktail (50 µM 2-amino-5-phosphonovalerate (APV), 40 µm 6-cyano-7-nitroquinoxaline-2,3-dione (CNQX) and 100 nM tetrodotoxin (TTX)) to block spontaneous activity for 3 h. Repetitive action potential firing was then induced by washing out the blockers (three changes of media) and adding 50 µM bicuculline (BiC) and 500 µM 4-aminopyridine (4-AP)) [23]. Transcription was blocked in one set of the cultures with 25 µm actinomycin D. In parallel, control cultures were treated with DMSO and maintained in the inhibitory cocktail. After 5 min, RNA was either harvested (5 min) or media was replaced with fresh media lacking BiC/4-AP.

To overcome some of these limitations, we devised a strategy to measure indirectly activity-dependent changes in transcript stability in hippocampal neurons. mRNA expression was measured by microarray analysis in hippocampal neurons under conditions in which transcription was blocked with the RNA polymerase II transcriptional inhibitor actinomycin D, whereas synaptic activity was increased for 5 min with bicuculline (BiC) and 4-AP treatment [23,24] (figure 1). In this paradigm, increased transcript abundance would correspond to mRNA stabilization and decreased transcript abundance would correspond to mRNA destabilization.

Post-transcriptional regulatory mechanisms that contribute to synaptic plasticity by altering stability of specific mRNA transcripts may be identified through a bioinformatics analysis of the 3′-UTRs of transcripts. Many of the motifs that were identified in these experiments were found to resemble mRNA transport sequences, RNA stability elements and microRNA (miRNA)-binding sites. Several of the corresponding miRNAs, including miR-326/miR-330, miR-485, miR-666 and miR-761 were found to be developmentally regulated in the hippocampus. Furthermore, one of these miRNAs, miR-485, predicted to affect central nervous system (CNS) morphogenesis and neurite outgrowth, controls nerve growth factor (NGF)-induced neurite outgrowth in PC12 cells. These motifs and corresponding binding factors may be an important regulatory mechanism for synaptic plasticity and neural development.

## 2. Material and methods

### (a) Animals

Timed-pregnant albino Sprague–Dawley rats were used throughout this study for hippocampal cell cultures. Timed-pregnant NIH Swiss mice were used for dorsal root ganglia cultures. Neonatal rats were euthanized by intraperitoneal injection of sodium pentobarbital, decapitated and the hippocampus was rapidly dissected in Pucks' D1 buffer and processed for total RNA.

### (b) Drug treatments and reagents

All drug treatments were made by replacing culture medium with fresh, pre-warmed and gassed sterile-filtered culture medium. Drugs were used at the final concentration (micromolar): bicuculline methiodide (BiC), 50; tetrodotoxin (TTX) Na^+^-citrate, 100 (Sigma, St. Louis, MO, USA); d-2-amino-5-phosphonovalerate (D-APV), 50; 6-cyano-7-nitroquinoxaline-2,3-dione (CNQX), 40; 4-aminopyridine (4-AP), 500 (Tocris Biosciences, Ellisville, MO, USA); actinomycin D, 25 (Life Technologies, Carlsbad, CA, USA); nerve growth factor (NGF), 50 ng ml^−1^.

### (c) Cell culture

Primary hippocampal neuron cultures were prepared from timed-pregnant Sprague–Dawley rats (E18.5). Hippocampi were dissected out in ice-cold Pucks' D1 buffer, and dissociated by trituration following incubation in 0.25% trypsin for 15 min at 37°C. Hippocampal cells were plated at either high density (2.6 × 10^4^ cells cm^−2^) atop a glial feeder layer for microarray and mRNA analysis or medium density (1.3 × 10^4^ cells cm^−2^) for immunocytochemistry. Cultures were plated on poly-l-lysine-coated (Sigma) coverslips in Neurobasal medium (Life Technologies) containing 2% B27, 25 µM glutamate, 2 mM GlutaMax, 100 U ml^−1^ penicillin, 100 μg ml^−1^ streptomycin and 10% FBS in a humidified atmosphere at 37°C and 5% CO_2_. The medium was replaced 2 h later with medium lacking glutamate and FBS; half-changes were made every 3–4 days. Hippocampal cultures were used for experiments at 12 days *in vitro* (DIV) at which time cultures had undergone extensive axonal and dendritic growth and formed many synaptic connections.

Dorsal root ganglion (DRG) neurons were dissociated from 13.5 days mouse embryos and plated at a density of 0.5 × 10^6^ cells ml^−1^ into each side compartment (250 μl per compartment) of Campenot chambers [25] in Eagle minimum essential medium with Earle's salts and supplements, containing 5% horse serum (HS) and 100 ng ml^−1^ NGF as described previously [26]. Non-neuronal cells division was inhibited by the addition of 13 μg ml^−1^ fluoro-2-deoxyuridine and uridine 1 day following plating for 4–5 days. Cultures were subsequently used for experiments 3–4 weeks after plating, at which time they display a mature axonal outgrowth.

Neuroscreen-1 cells (Thermo Scientific, Waltham, MA, USA) were maintained in RPMI medium containing 10% HS, 5% FBS, 2 mM GlutaMax, 100 U ml^−1^ penicillin, 100 μg ml^−1^ streptomycin in a humidified atmosphere at 37°C and 5% CO_2_. Following trypsinization, cells were re-plated at 1.22 × 10^4^ cells cm^−2^ on poly-l-lysine/collagen-coated coverslips (Advanced Biomatrix, San Diego, CA, USA) and grown for 24 h prior to transfection and induction of neurite outgrowth with 50 ng ml^−1^ NGF.

### (d) Activity protocol

In order to reduce basal transcriptional activity regulated by spontaneous activity, all hippocampal cultures were pre-incubated in Neurobasal (without B27, containing GlutaMax) in an inhibitor cocktail containing 50 µM D-APV, 40 µM CNQX and 100 nM TTX for 3 h. Cells were rinsed twice for 5 min with medium containing either 25 µM actinomycin D or DMSO control (0.1%) and pre-incubated for 15 min total in inhibitor cocktail. Cells were then treated with 50 µM BiC/500 µM 4-AP for 5 min or inhibitors, and mRNA was harvested either immediately following 5 m stimulation or at 30 min and 60 min post-stimulus. A short, 5 min stimulus was used in order to investigate rapid post-transcriptional changes in gene expression. For experiments with DRG neurons, media was exchanged for serum- and NGF-free medium overnight prior to electrical stimulation for 2 h as previously described [27].

### (e) Lipofectamine-mediated transfection and imaging

Hippocampal cultures at 7 DIV were co-transfected with 2 μg DsRed-C1 (Clontech, Mountainview, CA, USA) and either 25 pmol of miR-485-5p mimic, miR-485-5p inhibitor, miR negative controls, or distilled H_2_O (Ambion, Austin, TX, USA) and Lipofectamine 2000 (Life Technologies), as previously described [24]. miRNA mimics used were small, chemically modified, double-stranded molecules designed to act as endogenous miRNAs. The anti-miR inhibitors are chemically modified, single-stranded molecules designed to bind to and inhibit endogenous miRNA molecules similar to antisense. miRNA negative controls were designed to not either resemble known miRNAs (negative control for miR-mimic) or bind to and inhibit miRNAs (negative controls for miR inhibitor). For experiments on Neuroscreen-1 cells, 24 h following plating, cells were co-transfected with 25 pmol of miRNAs (as for hippocampal neurons) and 2 μg DsRed. Neurite outgrowth was induced by treatment with 50 ng ml^−1^ NGF 3 h following transfection. Neurite outgrowth index (NOI), calculated as the percentage of cells with neurites longer than twice the cell width was measured 96 h post-transfection. For analysis of miRNA effects on axons, 12 DIV cultures were fixed in 4% paraformaldehyde in PBS containing 4% sucrose and 10 mM EGTA for 30 min. Coverslips were then rinsed three times in PBS containing 4% sucrose, permeabilized in 0.1% Triton X-100 for 5 min, and free aldehydes were quenched with 50 mM NH_4_Cl and 50 mM glycine. Coverslips were blocked in 3% normal goat serum (Jackson ImmunoResearch) for 1 h and primary antibody incubations (anti-tau-1 clone PC1C6 at 1 : 1000 (EMD Millipore, Billerica, MA, USA), chicken anti-MAP2 at 1 : 2500 (EMD Millipore), mouse SMI-312 at 1 : 1000 (Covance, Princeton, NJ, USA) were performed overnight at 4°C. Highly cross-absorbed Alexa-conjugated secondary antibodies (Life Technologies) were incubated at 2 µg ml^−1^ for 2 h at room temperature. Coverslips were counterstained with Hoechst 33342 at a 1 : 5000 dilution for 5 min and mounted in Vectashield (Vector Labs, Burlingame, CA, USA). All images were acquired on a Zeiss laser scanning microscope 510 NLO from randomly selected neurons expressing DsRed with a 40 × (1.3 N.A.) oil-immersion lens using appropriate laser lines and excitation/emission filters.

### (f) Semi-quantitative RT-PCR

miRNA expression analysis in rat hippocampus was analysed by miRNA RT-PCR. Total RNA including non-coding RNA was purified using TRIzol (Life Technologies) aided by the addition of 1 µg of glycogen. RNA (2.5 µg) was poly-adenylated and reverse-transcribed using the NCode first strand cDNA synthesis kit (Invitrogen, Carlsbad, CA, USA). Transcript abundance, normalized to the snRNA U6, was analysed by real-time PCR on an ABI 7300 instrument (Applied Biosystems, Foster City, CA, USA).

### (g) Microarray and data analysis

Total RNA was extracted from DRG and hippocampal cell cultures using TRIzol reagent (Life Technologies). Microarray hybridizations were performed on two platforms: custom mammalian genome collection microarrays (GEO Platform GPL1211) and Illumina RatRef-12 Expression BeadChip arrays (Illumina, San Diego, CA, USA). Because changes in gene expression were measured following only 5 min of synaptic activity in the absence of transcription in hippocampal neurons, changes in the expression levels of genes were small and variable between both biological replicates as well as across microarray platforms. We therefore analysed individual microarray datasets. Furthermore, significant changes in expression levels were only considered for analysis where the *z*-fold expression change was more than |1.4| for both + and − actinomycin D datasets (co-regulated). This cut-off was then used to classify transcripts as either downregulated (destabilized) or upregulated (stabilized). For nylon membrane microarrays, 5 µg of total RNA pooled from two separate experiments for each condition was radiolabelled and hybridized to MGC nylon arrays containing approximately 16 800 individual clones as previously described [28], with the exception that RNAs isolated from DRG axon and cell body compartments were amplified using the MessageAmp II aRNA Amplification Kit (Ambion) prior to hybridization. Microarrays were exposed to phosphor imager screens for 12–24 h, scanned on a Molecular Dynamics Storm Phosphorimager (50 µm resolution) (GE Healthcare Bio-Sciences, Pittsburgh, PA, USA) and processed through either ImageQuant (GE Healthcare Bio-Sciences) or ArrayPro software (Media Cybernetics, Rockville, MD, USA). Raw hybridization intensity values were normalized using *z*-score transformation [29]; *z*-fold values ±1.4 were used as an arbitrary significance cut-off.

For Illumina BeadChip arrays, total RNA was further purified by RNeasy columns (Qiagen, Venlo, The Netherlands) and RNA quality was assessed on an Agilent 2100 Bioanalyzer (Agilent Technologies, Palo Alto, CA, USA). RNA samples were labelled according to the chip manufacturer's recommended protocols and quantified using an Illumina BeadStation 500GX Genetic Analysis Systems scanner and beadstudio software as previously described [30]. Background filtering of raw microarray data was adjusted by raising the *p*-value of detection from 0.01 to 0.1 in order to measure changes in modestly expressed genes [31]. This background filtering removed 55% of all the genes present on the Illumina microarray (12 479 background genes from among a total of 22 523 RefSeq genes). Overall differences in gene expression between samples were calculated by a global normalization method based on total intensity counts for each array. Normalized intensity values across samples were then *z*-transformed in order to calculate *z*-fold data [29]. The data for both microarray platforms are accessible through GEO series accession no. GSE55781.

Gene clusters were constructed using Cluster 3 software [32] and visualized using Java Treeview [33] by hierarchical clustering and complete linkage analysis. Gene ontology (GO) and pathway analysis was performed using Database for Annotation, Visualization and Integrated Discovery (DAVID) [34,35] (http://david.abcc.ncifcrf.gov/) and Ingenuity Pathway Analysis software (Ingenuity Systems, Redwood City, CA, USA). Four-way Venn diagrams were constructed to show either target gene or GOs enrichment using the program Venny (http://bioinfogp.cnb.csic.es/tools/venny/index.html).

### (h) 3′-UTR motif analysis

3′-UTRs for post-transcriptionally regulated genes were extracted from the UCSC Genome Browser [36,37] and analysed using standalone MEME and multiple alignment and search tool (MAST) software under Ubuntu 64 bit v. 13.10 [38,39]. The frequency of occurrence for any one motif either within a sequence set or within an individual 3′-UTR (anr, min and max sites) was adjusted to the input dataset size as recommended. Program settings were also adjusted in order to identify two classes of strand-specific *cis*-acting motifs: short fixed-width motifs (*n* = 8 nt) or variable-width motifs (7 ≤ *n* ≤ 15). These two motif sets were investigated because short defined motifs may either correspond (i) to miRNA-binding sites or (ii) core domains that form part of larger regulatory elements. Longer, variable-width motifs may also predict similar function to short motifs or map to less conserved types of elements that target RNAs to axonal and dendritic compartments. Only discovered motifs with *E*-values less than 0.1 were considered; motifs comprising long, low-complexity runs of single nucleotides were not included in the analysis. 3′-UTR datasets comprising non-regulated genes for all conditions and time points (|*z*-fold| < 1) were analysed in order to compare control motifs against post-transcriptionally enriched motifs.

Statistically significant motifs were analysed by MAST against a rodent 3′-UTR database consisting of 18 456 3′-UTRs extracted from the current Rat RefSeq database (March 2012, RGSC 5.0/rn5). Motifs with similarity scores of more than 0.6 were manually inspected and removed to improve MAST accuracy.

### (i) Data and statistical analysis

All values are reported as mean ± s.e.m. For miRNA analysis in neonatal animals, total RNA was pooled from at least two animals per replicate and normalized to transcript levels for snRNA U6. For immunocytochemistry, gain and offset settings were optimized for each fluorescent channel within a given experiment. For PC12 neurite outgrowth assays, NOI was calculated by measuring the cell diameter and length of neurites. PC12 cells with neurites longer than twice the cell body diameter were scored positive for neurite outgrowth. NOI was calculated from the mean of six fields of view and data are reported as *n* = 6 independent transfections. Descriptive statistics, including two-tailed Student's *t*-test, one-way ANOVA and post hoc tests (Dunnett's and Kruskal–Wallis) were used to assess statistical significance using Minitab (Minitab Inc., State College, PA, USA) and SigmaPlot v. 10.0 software (SPSS, Chicago, IL, USA).

## 3. Results

Transcripts regulated by synaptic activity (BiC/4-AP treatment) independently of transcription (during actinomycin D block) (figure 1) were profiled by microarray analysis (*z*-fold ≥ [1.4]). Fewer than 5% of the total genes were found to be post-transcriptionally regulated by increased neuronal activity for 5 min in hippocampal neurons. The types of biological processes associated with these mRNA transcripts differed depending on whether transcripts were destabilized or stabilized. Functional annotation clustering and pathway analysis of downregulated transcripts (410 genes) and upregulated transcripts (148 genes) showed enrichment for GOs associated with axon specification, synaptic vesicles and transcription (table 1). Transcripts that were not regulated by synaptic activity were enriched for pathways associated with protein synthesis and organelle function. More importantly, we found that many transcripts can be rapidly regulated by synaptic activity in a transcription-independent manner.

**Table 1:** Destabilized transcripts are enriched in GO terms associated with axonal development and synaptic vesicles.

condition	GO term	no. genes	*p*-value
downregulated (410)	neuron projection	31	9.1 × 10^−11^
	neuron differentiation	22	1.3 × 10^−4^
	synapse	22	1.9 × 10^−4^
	membrane-bound vesicle	23	1.9 × 10^−4^
	ion binding	77	2.1 × 10^−4^
	regulation of transcription	25	3.9 × 10^−4^
upregulated (148)	ribosome	6	2.5 × 10^−4^
	vesicle	12	1.6 × 10^−3^
	long-chain fatty acid transport	3	1.1 × 10^−2^
	nucleolus	6	2.7 × 10^−2^
non-regulated (136)	non-membrane-bounded organelle	28	2.3 × 10^−4^
	ribosome	6	2.6 × 10^−4^
	T-cell activation	5	1.9 × 10^−3^
	enzyme binding	11	2.1 × 10^−3^

GO analysis was performed on *z*-fold normalized microarray data from Illumina microarrays with a threshold of |*z*-fold| > 1.3 for both actinomycin D pre-treated and control cultures treated with BiC/4-AP for 5 min. Non-regulated transcripts were defined as those transcripts for all of the microarray samples where |*z*-fold| < 1. Top functions were identified from the DAVID [34,35] using functional annotation clustering and medium classification stringency. Downregulated or destabilized genes were mapped to functions and pathways important in nervous system development, e.g. neuron projection, synapse.

### (a) Axonal transcripts regulated by activity

In addition to being able to regulate gene expression rapidly, post-transcriptional regulation of mRNA stability can also provide critical subcellular regulation. This is particularly important in neurons which are complex, highly polarized cells, with distinct functions mediated in separate cellular compartments (dendrites, synaptic spines, cell body and axons). The axonal compartment is a difficult subcellular region to investigate in this regard, but the extremely long distance separating axon terminals from the cell nucleus would seem to make local regulation of mRNA levels especially advantageous to alter axon sprouting and outgrowth locally in response to electrical activity.

To test this hypothesis, we examined the findings on mRNA stability from experiments on hippocampal neurons in another preparation. Experiments on DRG neurons were undertaken because of the extremely long slender axons in these neurons (some up to 1 m in length). Secondly, DRG neurons lack dendrites, making them ideal for studies of the axonal and cell body compartments. The axonal compartment can be isolated from the cell body using multi-compartment cell cultures (Campenot chambers) [40] for biochemical and molecular biological analysis [41]. DRG axons can be stimulated through platinum electrodes in the cultures [25] and mRNA transcripts isolated from axons or cell bodies for analysis. Furthermore, electrical stimulation can be delivered in specific patterns and frequencies to investigate the dependence of mRNA transcript abundance (or second messenger activation and protein expression) on the specific pattern of neuronal firing [27,42].

**Figure 2.**
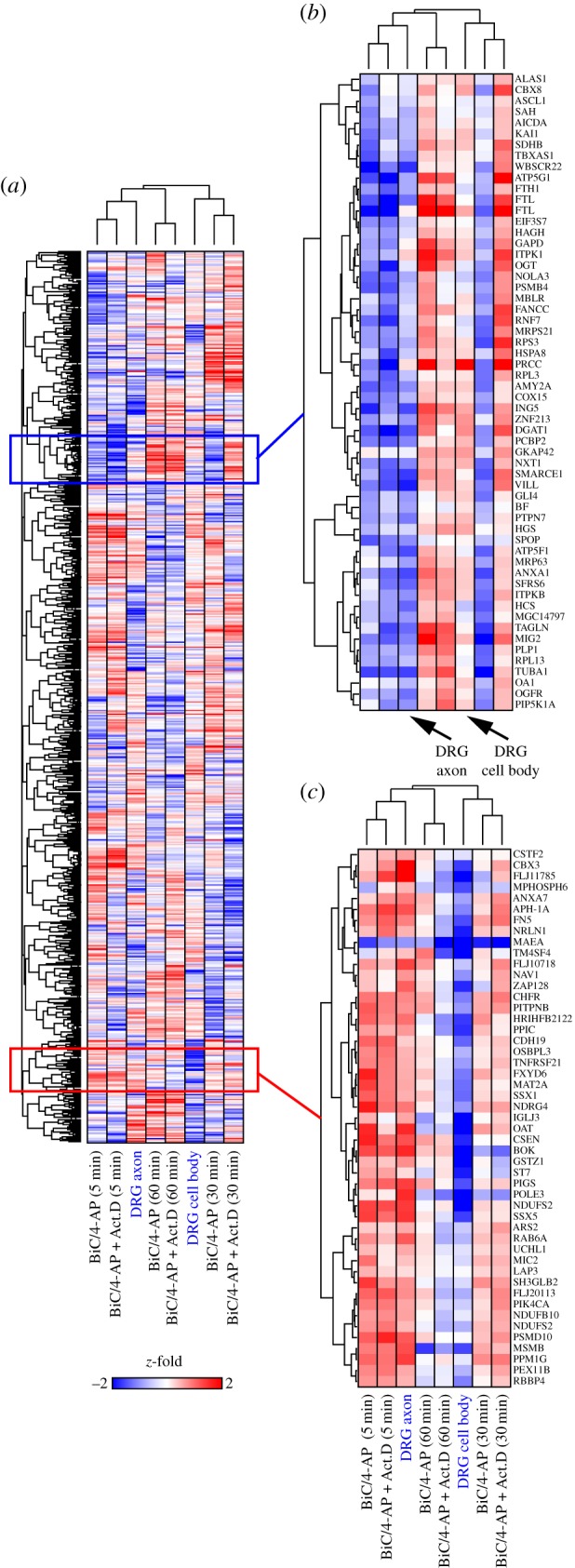
(*Opposite*.) Rapid, post-transcriptional regulation in hippocampal neurons mirrors activity-regulated genes in DRG axons. Hierarchical clustering of microarray expression data shows the regulation of hundreds of genes following neural activity in cultured hippocampal neurons and DRG neurons. These results were compared against gene expression changes in DRG neurons following 2 h of patterned electrical stimulation by *z*-fold normalized expression changes in post-transcriptional gene expression at 5, 30 and 60 min in hippocampal neurons (±actinomycin D) following a short synaptic stimulus (5 min BiC/4-AP). DRG neurons were electrically stimulated in multi-compartment chambers (electronic supplementary material, figure S1) in order to measure changes in gene expression in axons (DRG axons) and cell bodies (DRG cell bodies) separately. Unsupervised hierarchical clustering of microarray data from MGC nylon membranes is shown. In *b* (upregulated) and *c* (downregulated) are example clusters from *a*, further demonstrating co-regulation of transcripts in DRG axons and hippocampal neurons by a post-transcriptional mechanism.

We hypothesized that many of the transcripts that were found to be regulated in hippocampal neurons by increased synaptic activity during transcriptional blockade would also be regulated in the axonal compartment of DRG neurons because of the long distance of presynaptic terminals from the nucleus. Hierarchical clustering of *z*-fold normalized expression data [29] showed that transcripts enriched in DRG axons by electrical stimulation, clustered with transcripts enriched in hippocampal neurons treated with BiC/4-AP and actinomycin D (figure 2). Transcripts regulated by 2 h of electrical activity in DRG neurons would not have had time to be transcribed in the nucleus and transported several millimetres (more than 5 mm) into axons (electronic supplementary material, figure S1) [43,44]. These results support the hypothesis that local post-transcriptional regulation underlies rapid changes in hippocampal transcripts by 5 min and in the axonal compartment of neurons (figure 2; compare normalized *z*-fold levels in electrically stimulated DRG axons and BiC/4-AP-treated hippocampal neurons at 5 min).

This post-transcriptional regulation could affect axonal development, morphology, sprouting or other changes in presynaptic terminals. This question will be considered experimentally below, but first the question of how stability of specific mRNAs is regulated by neural firing will be addressed.

### (b) Analysis of 3′-UTRs of stabilized and destabilized transcripts

We next addressed how the stability of these mRNA transcripts was regulated by electrical activity. Our hypothesis was that specific RNA-binding sites would be found in the populations of mRNA transcripts that were selectively stabilized or destabilized by electrical activity. The 3′-UTRs of post-transcriptionally regulated genes were analysed using the MEME software suite [38] to identify two classes of *cis*-acting motifs: (i) short defined motifs corresponding to either miRNA-binding sites or core domains of larger regulatory elements and (ii) longer, less conserved motifs corresponding to mRNA stability and targeting elements. Motif analysis was performed on gene sets that were either stabilized or destabilized by synaptic activity and compared against the set of genes that were found not to be regulated by electrical activity. This approach assumes that these regulatory motifs would not be highly represented in the population of RNA transcripts that were not changed after neuronal stimulation.

### (c) Destabilized genes contain motifs corresponding to miRNA seed domains

**Figure 3.**
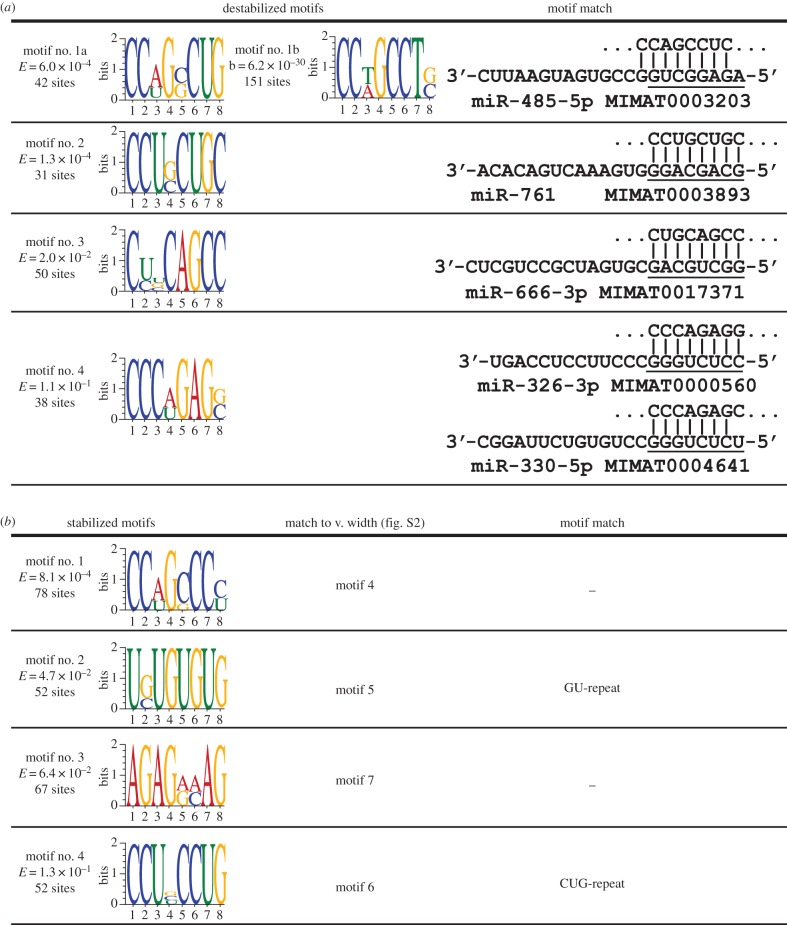
Discovery of enriched motifs for destabilized and stabilized genes. 3′-UTR analysis by MEME on downregulated (destabilized) and upregulated (stabilized) transcripts by MEME identifies miRNA-binding sites. Motifs from MEME analysis are shown for destabilized (*a*) and stabilized (*b*) datasets. 3′-UTR DNA sequences for regulated transcripts were retrieved from the rn5 assembly of the UCSC Genome Browser and analysed for motifs on the given strand for any number of repetitions, allowing for motifs of either an eight-base fixed width or a variable length between seven and 15 bases. The likelihood of discovery for the indicated motifs by chance is shown as an *E*-value. For each discovered motif, WebLogo plots [46] are shown as well as either a predicted miRNA seed match or match to variable-width motif (electronic supplementary material, figure S2). Only motifs with *E*-values less than 0.15 are reported.

We first investigated short, fixed-width motifs because miRNAs promote mRNA destabilization predominately through binding to the 3′-UTRs by targeting an eight nucleotide seed domain. These short motifs were searched against two miRNA databases, miRBase [19] and mESAdb [45]. We found that four of the discovered motifs (figure 3*a*) in the destabilized gene set corresponded to seed domains of miRNAs miR-326-3p/miR-330-5p, miR-485-5p, miR-666-3p and miR-761. Of the miRNA motifs that were identified, motifs corresponding to seed domains for miR-666-3p and miR-485-5p were over-represented in the variable-width motif set (electronic supplementary material, figure S2). Furthermore, analysis on a second microarray platform also independently showed enrichment for miR-485-5p (figure 3, motif 1b). Several GU- and CUG-repeats (e.g. [47]) were also identified that did not correspond to miRNA seed domains. By contrast, motifs that were over-represented in stabilized genes (figure 3*b*) did not correspond to either miRNA seed domains identified in destabilized gene sets or have corresponding miRNA sites. Motifs identified for non-regulated genes also lacked similarity to miRNA seed domains. MEME parameters were also adjusted in order to search for longer, variable-width motifs for destabilized and stabilized transcripts (electronic supplementary material, figure S2); these sites were distinct from the fixed-width sites found in the destabilized sets. Furthermore, five significant motifs identified for non-regulated transcripts were present in both destabilized and stabilized motifs sets. Motif enrichment for miRNA-binding sites preferentially in the destabilized set supports one of their functions: control of mRNA degradation [48] as well as our hypothesis that miRNAs contribute to rapid destabilization of transcripts.

### (d) Multiple alignment and search tool analysis of enriched motifs and pathway analysis

Statistically significant motifs from each dataset (*E*-value less than 0.1 and not comprising long, low-complexity nucleotide runs of single nucleotides) were analysed against a rat 3′-UTR database using MAST [39] in order to identify gene networks and pathways enriched for stabilizing and destabilizing motifs. MAST analysis of 3′-UTR motifs (figure 3) identified 107 transcripts that were enriched in GOs for embryonic development, cell projection and cytoskeleton (table 2 and the electronic supplementary material, table S1; destabilized 8 nt). However, only nine transcripts were identified by MAST that were enriched in stabilizing motifs. Analysis of the destabilized variable-width motif set (electronic supplementary material, figure S2) identified 1584 transcripts (*E*-value < 0.001) with GO and functional enrichment for embryogenesis, neuronal differentiation and axon guidance (table 2 and the electronic supplementary material, table S1; destabilized). Target prediction with the stabilized variable-width motif set, identified 2105 transcripts (*E*-value < 0.001) that had enrichment for ontology and function in cell projection, synapse and vesicle (table 2 and the electronic supplementary material, table S1; stabilized). By contrast, analysis of the motif set enriched in non-regulated genes, identified 391 transcripts that were predominately enriched in transcriptional regulation and gene expression (table 2 and the electronic supplementary material, table S1).

**Table 2.** MAST analysis of MEME predicted 3′-UTR motifs.

cluster	destabilized	score
1	transcriptional regulation	11.0
2	embryonic morphogenesis/development	8.5
3	vasculature development	8.4
4	neuron differentiation/morphogenesis/axon guidance	7.7
5	negative regulation of gene expression	7.2
6	nuclear/organelle lumen	5.9
7	regulation of cell motion/migration	5.5
8	limb morphogenesis/development	5.5
cluster	stabilized	score
1	transcriptional regulation-positive	8.9
2	transcriptional regulation	7.0
3	cell projection/dendrite/soma	5.9
4	membrane fraction	5.3
5	cytoplasmic vesicle	5.0
6	protein dimerization/binding	4.9
7	synapse/post-synaptic membrane	4.8
8	embryonic development	4.7
cluster	non-regulated	score
1	transcriptional regulation	6.8
2	vasculature development	3.8
3	cell migration/motility	3.7
4	nuclear/organelle lumen	3.6
5	negative regulation of gene expression	3.4
6	embryonic development	2.9
7	skeletal development	2.8
8	embryonic morphogenesis/development	2.4
cluster	destabilized (8nt)	score
1	embryonic morphogenesis/development	2.0
2	cell projection/cytoskeleton	1.8
3	protein dimerization/binding	1.7
4	transcriptional regulation	1.7
5	post-synaptic membrane/synapse	1.6
6	embryonic organ development/morphogenesis	1.5
7	cell differentiation	1.5
8	reproduction	1.5

GO analysis was performed on MAST predictions for destabilized and stabilized motif sequence. Top functions for each GO cluster were identified using DAVID functional annotation tools. Predicted transcripts were mapped to functions and pathways important in development, morphogenesis and cell polarization. GO enrichment for transcriptional regulation was present for all three conditions. MAST predictions with an *E*-value < 0.001 for variable-width motifs or *E*-value < 1 for fixed-width motifs (90 DAVID IDs or 107 transcripts) were analysed by DAVID. In total, 1360 DAVID IDs (1584 transcripts) were analysed for destabilized motif set, 1761 DAVID IDs (2105 transcripts) for stabilized set and 331 DAVID IDs (391 transcripts) for the non-regulated set. Calculated group enrichment scores for each GO cluster were calculated from the geometric mean (-log scale) of the *p*-value in each corresponding annotation cluster to rank biological significance. Top ranked annotation groups (highest score) likely have lower *p*-values for their annotated members.

### (e) Prediction of miRNA targets and gene ontologies enrichment for discovered miRNA motifs

**Figure 4.**
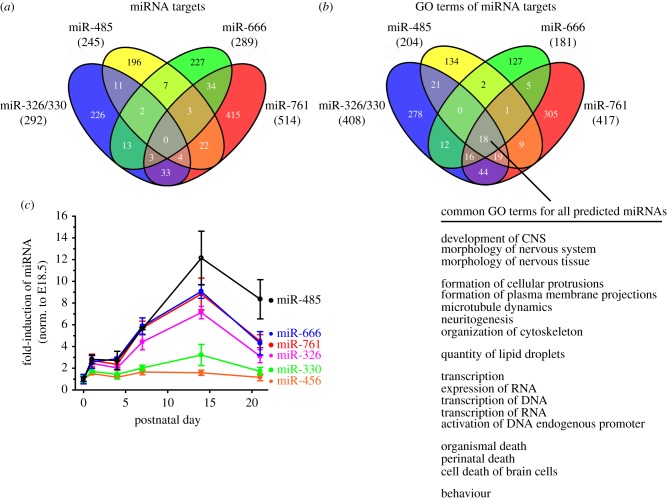
Predicted miRNAs are developmentally regulated in the hippocampus and may control nervous system development. (*a*) Venn diagram of miRNA target predictions (TargetScan v. 6.2) for miR-326-3p/330-5p, miR-485-5p, miR-666-3p and miR-761. Less than 15% of predicted targets are shared between any two miRNAs. The numbers in parentheses refer to the number of annotated genes predicted to contain at least one conserved miRNA-binding site. Specific targets are given in the electronic supplementary material, table S2. (*b*) Venn diagram of GO analysis for miRNA target predictions given in the electronic supplementary material, table S2. The number in parentheses refers to the number of significant GO terms with *p*-value ≤ 0.01. Eighteen GO terms were found to be enriched for all four miRNAs investigated, showing enrichment for nervous system development and morphogenesis. (*c*) Predicted miRNA transcripts were developmentally regulated in the hippocampus. The increase in miRNA transcripts from embryonic day 18.5 (E18.5) through postnatal day 21 (P21) (*n* = 3 animals at each time point) were first normalized to the reference gene U6 snRNA and the abundance expressed relative to E18.5. Fold induction of miRNAs varied from threefold to 12-fold.

We identified several motifs that resembled miRNA-binding sites within the 3′-UTRs of destabilized genes (figure 3 and the electronic supplementary material, figure S2). We therefore asked whether these predicted miRNAs may target gene networks that affect axonal development and presynaptic function. Target prediction and GO analysis of miR-326-3p/330-5p, miR-485-5p (miR-485-5p/1698/1703/1962), miR-666-3p and miR-761-5p showed enrichment for terms associated with synaptic transmission, plasticity, neurotransmitter synthesis and vesicular release, in both miRNA targets (figure 4*a*) and GO terms (figure 4*b* and table 3; and the electronic supplementary material, table S2). There was no overlap between all of the predicted miRNAs and their corresponding targets; however, 18 GO terms enriched in morphogenesis were identified, which suggested that the miRNAs may affect a common phenotype, such as nervous system development.

**Table 3:** Top networks and pathways of miRNA predicted targets identified by ingenuity pathway analysis.

miRNA	network or pathway	no. genes	*p*-value
miR-326-3p/330-5p (292)	neurological disease	61	9.34 × 10^−5^ – 1.38 × 10^−2^
	cell morphology	88	1.15 × 10^−8^ – 1.38 × 10^−2^
	CNS development and function	86	2.81 × 10^−9^ – 1.38 × 10^−2^
	Wnt/B-catenin		4.67 × 10^−6^
	actin cytoskeleton		4.43 × 10^−5^
	axonal guidance		3.24 × 10^−4^
miR-485-5p (245)	neurological disease	51	2.32 × 10^−5^ – 1.57 × 10^−2^
	cell morphology	61	1.4 × 10^−4^ – 1.51 × 10^−2^
	CNS development and function	61	6.75 × 10^−7^ – 1.57 × 10^−2^
	circadian rhythm signalling		6.74 × 10^−4^
	agrin interactions at neuromuscular junction		1.27 × 10^−3^
	axonal guidance signalling		1.36 × 10^−2^
miR-666-3p (289)	developmental disorder	53	1.11 × 10^−4^ – 1.63 × 10^−2^
	gene expression	71	9.13 × 10^−7^ – 1.62 × 10^−2^
	CNS development and function	65	3.69 × 10^−5^ – 1.89 × 10^−2^
	protein kinase A signalling		3.09 × 10^−3^
	synaptic long-term potentiation		7.2 × 10^−3^
	dopamine-DARPP32 feedback in cAMP		8.65 × 10^−3^
miR-761 (514)	cancer	256	5.07 × 10^−5^ – 1.32 × 10^−2^
	cell morphology	97	5.53 × 10^−8^ – 1.36 × 10^−2^
	CNS development and function	118	1.11 × 10^−7^ – 1.36 × 10^−2^
	axon guidance signalling		6.93 × 10^−4^
	RAN signalling		7.63 × 10^−4^
	netrin signalling		2.97 × 10^−3^

miR-326/miR-330 top canonical pathways.

Wnt/B-catenin: BTRC, CDH3, CSNK2A1, FZD4, FZD5, MARK2, NLK, PPP2R5B, RARG, SOX12, TCF4, TLE3.

Actin cytoskeleton: ABI2, F2R, FGD3, FGF9, FGF11, ITGA5, MAPK1, NRAS, PIP4K2C, SSH2, SOS1, TLN.

Axonal guidance: ADAM19, C9orf3, EFNA3, EPHB3, FZD4, FZD5, ITGA5, ITSN1, MAPK1, NGFR, NTN1, NRAS, PDIA3, SEMA3G, SRGAP3, SOS1.

miR-485 top canonical pathways.

Circadian rhythm signalling: GRIN1, ADCYAP1R1, CRY2, CREB1.

Agrin interactions at NMJ: PAK4, PAK1, DAG1, ACTG1, ITGAL.

Axonal guidance signalling: ARHGEF15, BAIAP2, EFNB3, PAK1, PAK4, PLCD3, PLXNA2, PFN2, PPP3R1, SEMA4G, SRGAP2.

miR-666-3p top canonical pathways.

Axon guidance signalling: AKAP6, CALM1 (includes others), CAMK2G, CREB5, EYA3, GNG12, PDE7A, PPP1CA, PPP1R10, PTPN2, RAP1B, TCF4, YWHAQ.

Synaptic long-term potentiation: CALM1 (includes others), CAMK2G, CREB5, PPP1CA, PPP1R10, RAP1B.

Dopamine-DARPP32 feedback in cAMP: CALM1 (includes others), CACNA1D, CSNK1G1, PPP1R10, CREB5, PPP1CA, DRD2.

Ingenuity pathway analysis was performed on predicted targets of miR-326-3p/miR-330-5p, miR-485-5p, miR-666-3p and miR-761 using the TargetScan v. 6.2 algorithm [49–51]. Predicted targets were mapped to specific biological functions and pathways: (i) disease and disorders, (ii) molecular and cellular functions, (iii) physiological system development and function, and (iv) top canonical pathways. Ingenuity's function analysis identified several biological functions and diseases most significant to genes within a network. The probability that a function or pathway was due to chance alone or enriched was determined by a Fisher's exact test. The top functions are shown, demonstrating that enriched miRNA sites are predicted to regulate morphogenesis, neural development and synapse formation. The complete list of predicted miRNA targets is given in the electronic supplementary material, table S2. The *p*-values in the range shown were considered highly significant.

miRNAs that act to control neural development and synaptic plasticity would be expressed in an activity- and time-dependent manner. We therefore tested whether the predicted miRNAs were expressed in the hippocampus and regulated in a developmental manner. Transcript levels for miR-326-3p, miR-485-5p, miR-666-3p and miR-761 increased by threefold to 12-fold by 14 days post parturition (figure 4*c*) (*n* = 3 animals per time point). Expression levels for miR-330-5p only modestly increased by 21 days post parturition (miR-456 expression, corresponding to motif 3 and sharing a seed motif with miR-485-5p was very low at all time points measured). We found that these miRNAs were expressed in the hippocampus in a developmental manner and that their predicted targets were enriched for functions important in pre- and post-synaptic development and plasticity. These miRNAs either individually or together, may act as a regulatory network in axonal outgrowth and presynaptic terminal formation during synapse formation. We therefore investigated the function of one of these miRNAs, miR-485-5p, on neurite outgrowth.

### (f) miR-485 regulates neurite outgrowth in PC12 cells

**Figure 5.**
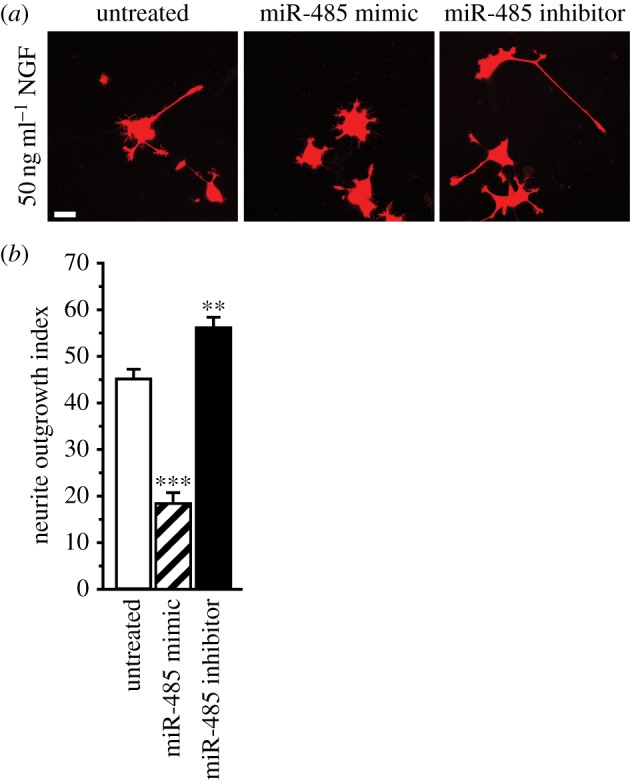
miR-485 blocks NGF-induced neurite outgrowth in PC12 cells. miR-485 overexpression inhibits NGF-induced neurite outgrowth in PC12 cells. Shown in (*a*) are representative examples of PC12 cells treated with 50 ng ml^−1^ NGF and co-transfected with DsRed and miR-485 mimic, miR-inhibitor and negative controls, and analysed at 96 h. Scale bar, 25 μm. Neurite outgrowth was inhibited by overexpression of a miR-485-5p mimic (*p* < 0.001) and enhanced by a miR-485 inhibitor (*p* < 0.001). PC12 cells with neurites longer than twice the cell body diameter were scored positive for neurite outgrowth (NOI). The NOI in untreated PC12 cells was 0.45 ± 0.02; treatment with negative controls for the mimic (-co. miR-M, 0.44 ± 0.04) or inhibitor (-co. miR-I, 0.43 ± 0.03) did not affect the NOI. *n* = 6 for all tested conditions. Effects of miR-M and miR-I on NOI were highly significant by one-way ANOVA (*F*_25,4_ = 26.15, *p* < 0.001). Overexpression of either a negative control for the miRNA precursor or the inhibitor did not significantly affect the NOI. Scale bar, 25 μm; **p < 0.01; ***p < 0.001.

We tested the function of miR-485-5p on NGF-dependent neurite outgrowth in PC12 cells, a well-established model of neurite outgrowth. Consistent with predicted function of miR-485-5p based on target analysis and GO enrichment for regulation of neuritogenesis and cytoskeletal organization (figure 4*b*), miR-485-5p overexpression dramatically reduced NGF-dependent neurite outgrowth (figure 5*a*,*b*, *p* < 0.001, *n* = 36 fields from six transfections). Conversely, transfection with a miR-485 inhibitor significantly increased neurite outgrowth (*p* < 0.01). Overexpression of either miR-mimic or miR-inhibitor negative controls did not affect NGF-induced neurite outgrowth. Effects of miR-485 on NOI were highly significant by one-way ANOVA (*F*_4,25_ = 26.15, *p* < 0.0001).

**Figure 6.**
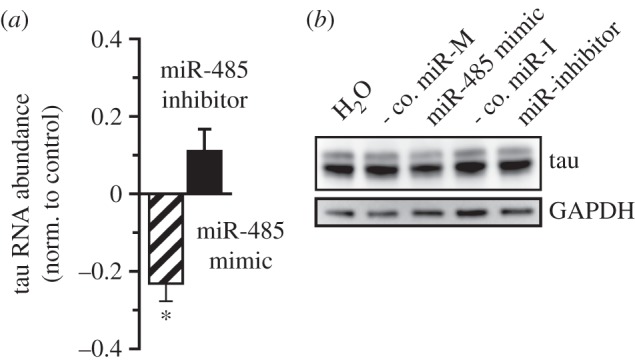
miR-485 decreases tau expression in hippocampal neurons. Overexpression of miR-485-5p modestly decreased tau transcript abundance (*a*) and protein levels (*b*) in hippocampal neurons. Cultures were transfected with miRNAs at 7 days *in vitro* (DIV) and analysed at 12 DIV. Fold change in transcript abundance for miR-overexpression and inhibition were normalized to their respective negative controls (negative control for either the mimic or inhibitor). Tau protein levels decreased relative to total GAPDH protein expression. Treatment with negative controls for either the mimic or inhibitor did not alter transcript levels for tau protein. *n* = 3, *p* < 0.05.

**Figure 7.**
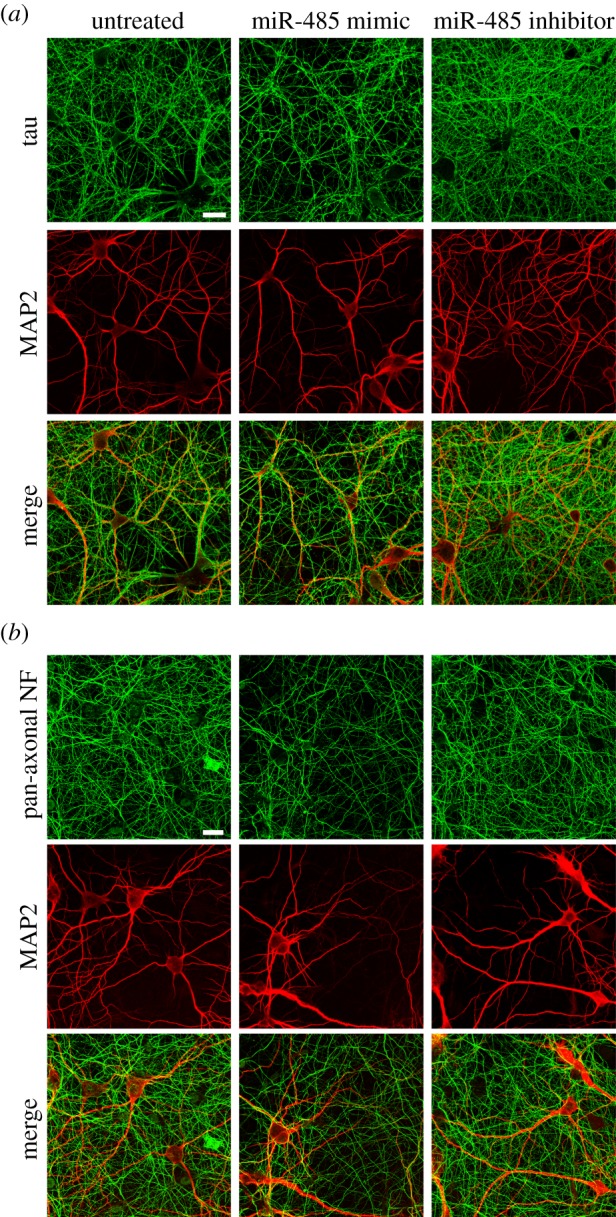
miR-485 overexpression controls tau expression and axonal outgrowth in hippocampal neurons. miR-485-5p overexpression reduced the extent of axonal outgrowth in hippocampal neurons. Cultures were co-transfected with DsRed and miRNAs at 7 DIV and analysed by immunocytochemistry at 12 DIV for axons by either tau or the pan-axonal neurofilament marker, SMI312. Dendritic staining was assessed by immunostaining for MAP2. Shown are representative examples of untreated (control) and neurons transfected with either a miRNA mimic or inhibitor showing that axonal outgrowth is regulated by miR-485-5p. Treatment with negative controls for either a miRNA mimic or inhibitor did not alter either axonal or dendritic staining. Double labelling with axonal and dendritic markers showed little co-localization. Scale bar, 25 μm.

The function of miR-485-5p on axons was next tested in hippocampal neurons. Overexpression of the miRNA reduced the expression of tau, measured by RT-PCR and western blotting (figure 6). Furthermore, miR-485-5p overexpression reduced the extent of axonal outgrowth in hippocampal neurons as measured by immunoreactivity to tau protein (figure 7*a*) and the pan-axonal neurofilament marker SMI312 (figure 7*b*). Dendritic staining for MAP2 was not affected by either miRNA overexpression or inhibition. The effects on miR-485-5p on both neurite outgrowth in PC12 cells and axonal markers further support a function of this miRNA on axonal development.

## 4. Discussion

These studies show that the abundance of specific mRNAs in hippocampal neurons changes within minutes of increasing synaptic activity. Post-transcriptional mechanisms, primarily those regulating the rate of mRNA degradation, are responsible. Moreover, many of these same mRNA transcripts are regulated in the axonal compartment of DRG neurons (electronic supplementary material, figure S1) by induced action potential firing (figure 2). Analysis of the 3′-UTRs of destabilized transcripts revealed an enrichment of motifs that correspond to miRNA seed domains (figure 3 and the electronic supplementary material, figure S2). miRNAs primarily function to promote mRNA degradation and translational suppression [48]. Post-transcriptional control within the time frame of several minutes could therefore occur through miRNA-mediated mRNA decay and contribute to local regulation of mRNA stability at the synapse. Moreover, analysis of the 3′-UTRs of either stabilized or non-regulated transcripts did not identify short, conserved motifs corresponding to miRNA seed domains (figure 3), further supporting these findings. By contrast, when 3′-UTR sequences were analysed for longer motifs (electronic supplementary material, figure S2), specifically for stabilized transcripts, several motifs were identified that may contribute to either mRNA transport or stabilization. We found that many of the motifs when searched against a rodent 3′-UTR database (table 2), were enriched in the 3′-UTRs of many transcripts important in axon guidance, development, plasticity and synapses. A number of predicted motifs (motifs 1–5; electronic supplementary material, figure S2) and MAST predicted transcripts (electronic supplementary material, table S1) were present in all three categories (stabilized, destabilized and not regulated); these motifs and corresponding RNAs may not necessarily be regulated in either an activity or developmental manner but be reliably expressed in hippocampal neurons. Alternatively, these longer motifs may correspond to either binding sites for RNA-BPs or translation factors.

Several of the miRNAs uncovered in this study (figure 4) were found to increase in hippocampus during postnatal development, consistent with possible involvement in nervous system formation and remodelling. More importantly, this study identified several miRNAs whose predicted targets are important in many aspects of nervous system development and expressed in the developing hippocampus. Many of the miRNAs that were identified are encoded within the introns of host genes that are expressed in hippocampus: miR-326 (Arrb1, β-arrestin), miR-330 (Eml2, echinoderm microtubule associate protein like 2) and miR-761 (Nrd1, Nardilysin) (electronic supplementary material, figure S3). Moreover, miR-485 and miR-666 are intronic miRNAs that are part of the large Dlk1-Dio3 imprinted locus [52,53] containing at least 53 miRNAs expressed in the CNS [54]. Fiore *et al*. [55] had previously shown that several of these miRNAs within the miR-379-410 cluster are co-regulated in response to neuronal depolarization and brain-derived neurotrophic factor, affecting dendritogenesis.

Regulation of mRNAs detected in the axonal compartment of DRG neurons after inducing action potential firing (figure 2) indicates subcellular control of transcript abundance that could be translated into plasticity-related proteins locally within the axonal compartment of neurons. One of the miRNAs identified here, miR-485-5p, was shown to regulate NGF-induced neurite outgrowth in PC12 cells (figure 5) and axonal development in hippocampal neurons (figures 6 and 7). We previously reported on the function of miR-485-5p on homeostatic changes in synapses through action on the presynaptic vesicle protein SV2A [24]. These findings further expand on the function of this miRNA.

In summary, these results identify several miRNAs that may function as important regulators of neural development and plasticity of the hippocampus. miRNAs are unique in their ability to coordinatively regulate large networks of mRNAs within distinct subcellular compartments. Of the approximately 2000 miRNAs that have been identified so far in rodents [19], the majority have not been characterized. Further analysis is needed in order to characterize these miRNAs *in vitro* and *in vivo* and their contribution to development and plasticity.

## Supplementary Material

Supplementary figures and references

## Supplementary Material

Supplemental Table 1: MAST predictions for significant motifs

## Supplementary Material

Supplemental Table 2: Summary table of predicted miRNA targets

## Notes

All procedures conformed to NIH animal welfare guidelines and approved animal study protocols.
