# Supplementary material for: Systematic identification of 3′-UTR regulatory elements in activity-dependent mRNA stability in hippocampal neurons
Source: Philos Trans R Soc Lond B Biol Sci. 2014 Sep 26;369(1652):20130509. doi: 10.1098/rstb.2013.0509 (PMC4142030; doi:10.1098/rstb.2013.0509)
Supplement: Supplementary figures and references [file rstb20130509supp1.pdf]

Phil. Trans. R. Soc. B. doi: 10.1098/rstb.2013.0509

SUPPLEMENTAL INFORMATION

Systematic identification of 3'UTR regulatory elements in activity-dependent mRNA stability in hippocampal neurons

Jonathan E. Cohen[*#], Philip R. Lee[*], and R. Douglas Fields[*]

*Section on Nervous System Development and Plasticity, Building 35, Room 2A211. The Eunice Kennedy Shriver National Institute of Child and Human Development. National Institute of Health, Bethesda MD 20892-3714.

#Current Address
Laboratory of Respiratory and Special Pathogens, Building 29, Room 111, Center for Biologics Evaluation and Research. Food and Drug Administration, Bethesda MD 20892

© 2014 The Authors under the terms of the Creative Commons Attribution License http://creativecommons.org/licenses/by/3.0/, which permits unrestricted use, provided the original author and source are credited.

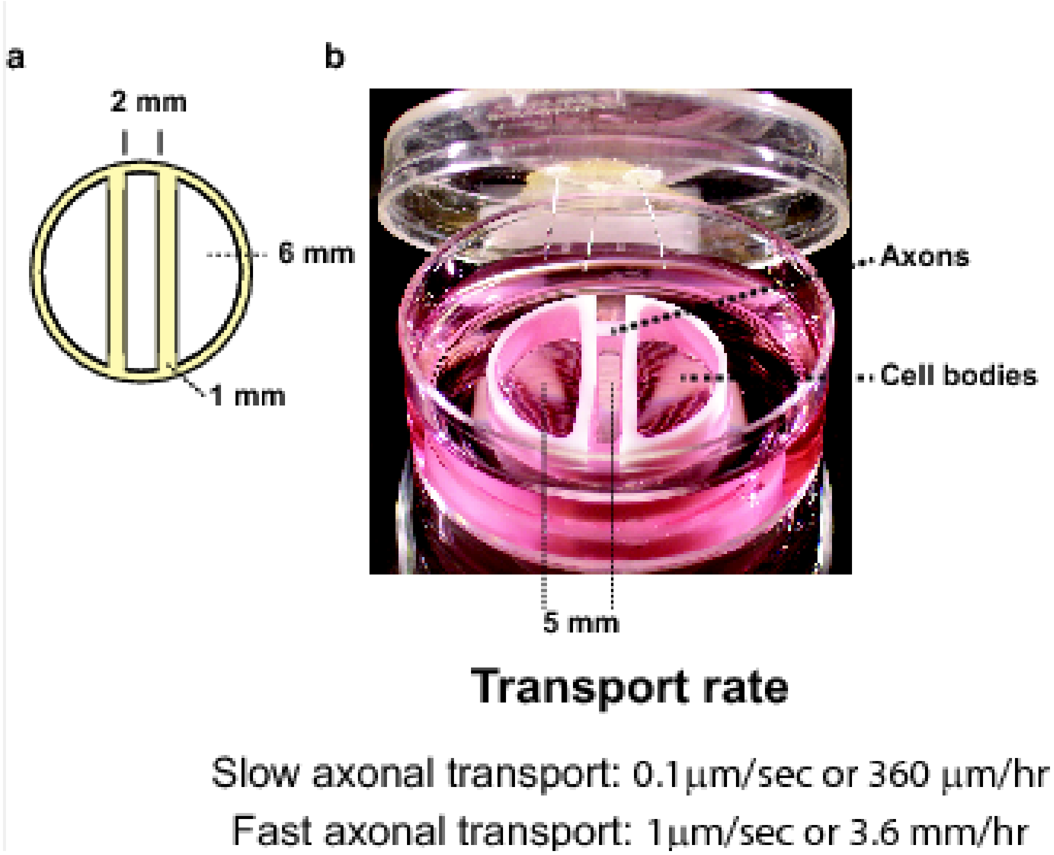

Supplementary Figure 1. Schematic of compartmentalized DRG neuronal cultures. Cultured dorsal root ganglia neurons were cultured in three-compartment Campenot chambers. DRG neurons were plated in the side compartments and maintained in culture for several weeks at which time the central compartment is enriched in a pure population of axons. Visual inspection is performed prior to electrical stimulation to ensure the absence of cell bodies within the central compartment. Assuming a mRNA transport rate of up to 1 µm/sec or 3.6 mm/hour and an average distance of 5 mm, little transcript will have been transcribed in the nucleus (side compartment) and transported under the barrier into the central compartment by two hours of electrical stimulation. Changes in gene expression in the axonal compartment, measured by microarray analysis, will have likely been due to changes in mRNA stability (similar to 5 min of synaptic activity in hippocampal neurons).

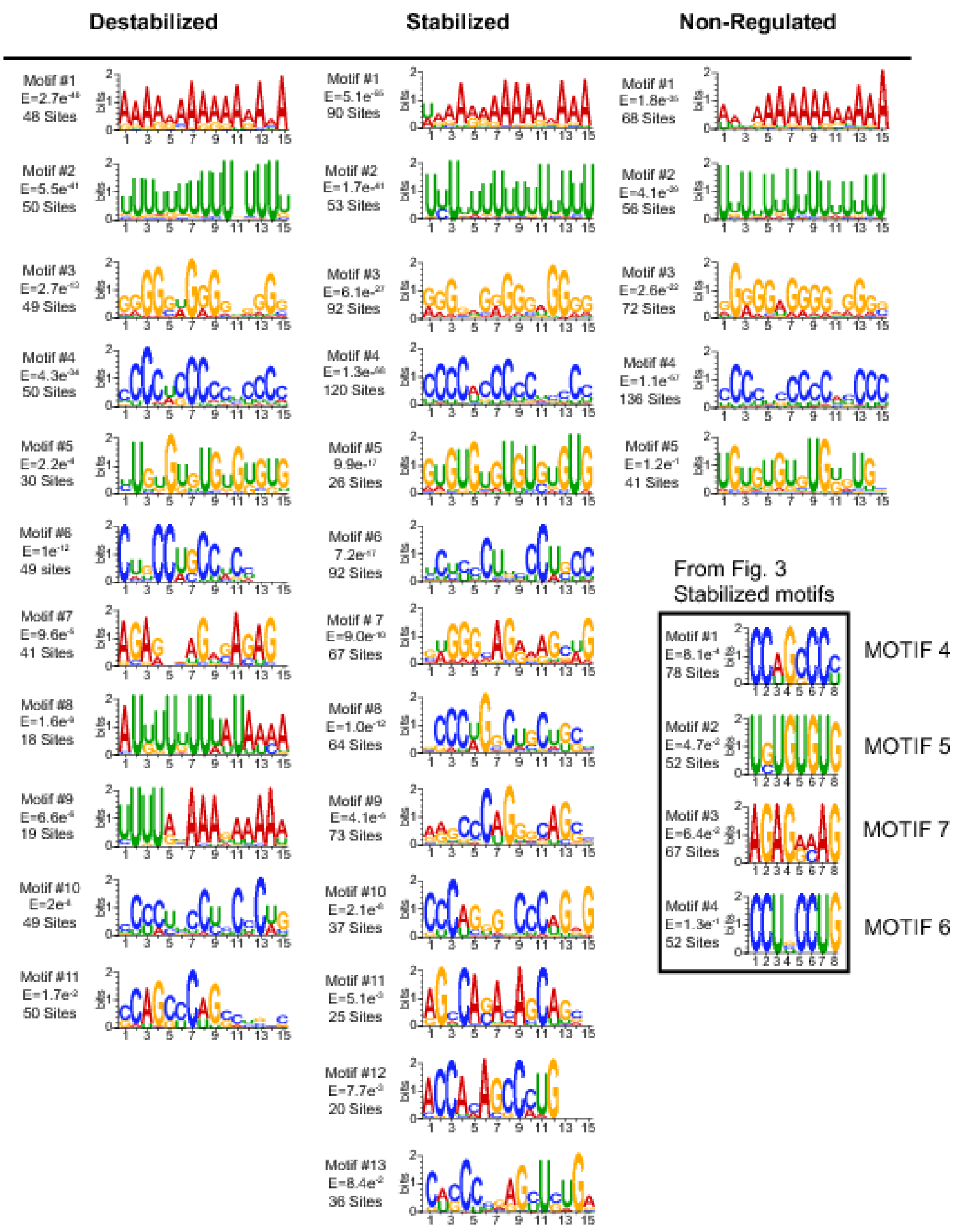

Supplementary Figure 2. Variable width motifs for non-regulated, destabilized and stabilized genes.

3’-UTR analysis was using the MEME software analysis package [1, 2] with parameters adjusted for variable-width motifs (7 – 15 nt) on non-regulated, destabilized, and stabilized transcripts. Significant motifs from MEME analysis are shown for destabilized, stabilized, and non-regulated datasets. All of the motifs identified for non-regulated transcripts matched motifs found in regulated transcript sets. Fixed width motifs identified for stabilized transcripts matched several of the variable-width motifs (MEME predicted motifs 4 through 7). 3’-UTR DNA sequences for regulated transcripts were retrieved from the rn5 assembly of the UCSC Genome Browser [3, 4] and analyzed for motifs on the given strand for any number of repetitions, allowing for variable length between 7 and 15 bases. The likelihood of discovery for the indicated motifs by chance is shown as an E-value. For each discovered motif, WebLogo plots [5] are shown as well as either a predicted miRNA seed match or match to variable-width motif (Fig S2). Only motifs with E-values less than 0.15 are reported.

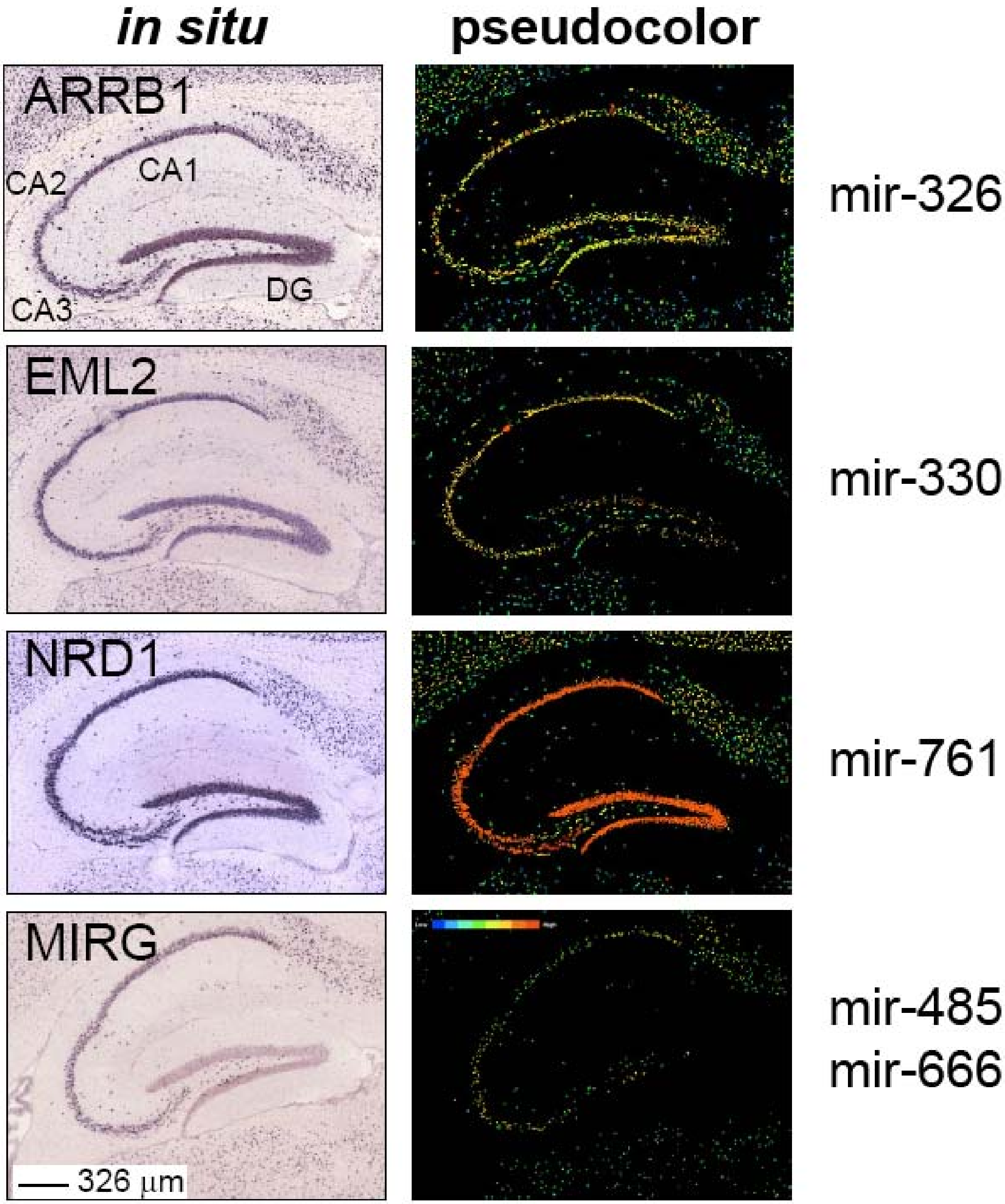

Supplementary Figure 3. *In situ* expression of miRNA host genes in mouse hippocampus. Representative *in situ* hybridization data from Allen Mouse Brain Atlas [6] (http://mouse.brain-map.org/) for miRNA present in the introns of host genes. Shown are *in situ* and pseudocolor images of sagittal sections for Arrbb1 (miR-326), EML2 (miR-330), Nrd1 (miR-761), and Mirg (miR-485 and miR-666). Varying levels of expression can be observed for the host genes, with modest expression of Mirg and high levels of expression for Nrd1 in the hippocampus. Scale bar = 326 μm.

Supplemental References

[1] Bailey, T.L. & Elkan, C. 1994 Fitting a mixture model by expectation maximization to discover motifs in biopolymers. *Proceedings / ... International Conference on Intelligent Systems for Molecular Biology ; ISMB. International Conference on Intelligent Systems for Molecular Biology* **2**, 28-36.

[2] Bailey, T.L. & Gribskov, M. 1998 Combining evidence using p-values: application to sequence homology searches. *Bioinformatics* **14**, 48-54.

[3] Karolchik, D., Hinrichs, A.S., Furey, T.S., Roskin, K.M., Sugnet, C.W., Haussler, D. & Kent, W.J. 2004 The UCSC Table Browser data retrieval tool. *Nucleic acids research* **32**, D493-496. (doi:10.1093/nar/gkh103).

[4] Kent, W.J., Sugnet, C.W., Furey, T.S., Roskin, K.M., Pringle, T.H., Zahler, A.M. & Haussler, D. 2002 The human genome browser at UCSC. *Genome research* **12**, 996-1006. (doi:10.1101/gr.229102. Article published online before print in May 2002).

[5] Crooks, G.E., Hon, G., Chandonia, J.M. & Brenner, S.E. 2004 WebLogo: a sequence logo generator. *Genome research* **14**, 1188-1190. (doi:10.1101/gr.849004).

[6] Lein, E.S. & Hawrylycz, M.J. & Ao, N. & Ayres, M. & Bensinger, A. & Bernard, A. & Boe, A.F. & Boguski, M.S. & Brockway, K.S. & Byrnes, E.J., et al. 2007 Genome-wide atlas of gene expression in the adult mouse brain. *Nature* **445**, 168-176. (doi:10.1038/nature05453).

Supplemental Tables supplied separately
(at http://rstb.royalsocietypublishing.org/lookup/doi/10.1098/rstb.2013.0509)

Supplemental Table 1 MAST predictions for significant motifs
Summary information is given for predicted transcripts from MAST analysis of (a) fixed-width motifs (5 MIN DOWN 8 NT MOTIF), (b) destabilized variable-width motif (5 MIN DOWN VAR), (c) stabilized, variable-width motifs (5 MIN UP VAR), or (d) NO REGULATION. MAST predictions with an E-value < 0.1 for fixed-width motifs or an E-value < 0.001 for variable-width motifs are given as a reference GenBank accession number and corresponding E-value for each transcript. The E-value given in the table is derived from the combined p-value of the entire 3'-UTR sequence, dependent upon the number of significant motifs identified for each candidate target 3'-UTR.

Supplemental Table 2 Summary table of predicted miRNA targets
miRNA target predictions for miR-326-3p/miR-330-5p, miR-485-5p, miR-666-3p, and miR-761 were performed using the TargetScanv6.2 algorithm. For all miRNA predictions with the exception of miR-761 (Mouse miR-214/761/3619-5p), rat was chosen as the species. Predicted targets with only site conservation in several species are listed in the table. For miR-326-3p/miR-330-5p and miR-214/miR-761 which have conserved seed domains, the combined predictions are given. Summary gene information (including GO headings) for each representative human GenBank accession number is given when available.
